# Extended Metabolic Biosensor Design for Dynamic Pathway Regulation of Cell Factories

**DOI:** 10.1016/j.isci.2020.101305

**Published:** 2020-06-23

**Authors:** Yadira Boada, Alejandro Vignoni, Jesús Picó, Pablo Carbonell

**Affiliations:** 1 Synthetic Biology and Biosystems Control Lab, I.U. de Automática e Informática Industrial (ai2), Universitat Politècnica de València, Camí de Vera S/N, 46022 Valencia, Spain; 2 Centro Universitario EDEM, Escuela de Empresarios, Muelle de la Aduana s/n, La Marina de València, 46024 Valencia, Spain

**Keywords:** Bioengineering, Metabolic Engineering, Biotechnology

## Abstract

Transcription factor-based biosensors naturally occur in metabolic pathways to maintain cell growth and to provide a robust response to environmental fluctuations. Extended metabolic biosensors, i.e., the cascading of a bio-conversion pathway and a transcription factor (TF) responsive to the downstream effector metabolite, provide sensing capabilities beyond natural effectors for implementing context-aware synthetic genetic circuits and bio-observers. However, the engineering of such multi-step circuits is challenged by stability and robustness issues. In order to streamline the design of TF-based biosensors in metabolic pathways, here we investigate the response of a genetic circuit combining a TF-based extended metabolic biosensor with an antithetic integral circuit, a feedback controller that achieves robustness against environmental fluctuations. The dynamic response of an extended biosensor-based regulated flavonoid pathway is analyzed in order to address the issues of biosensor tuning of the regulated pathway under industrial biomanufacturing operating constraints.

## Highlights

• Fluctuations and perturbations affect dynamics and titers in cell factories
• Extended metabolic biosensors expand product sensing and enable pathway regulation
• Closed-loop pathway regulation provides robustness to cell factory production
• A closed-loop regulated flavonoid production pathway shows robust dynamic response

## Graphical Abstract

**Figure:**
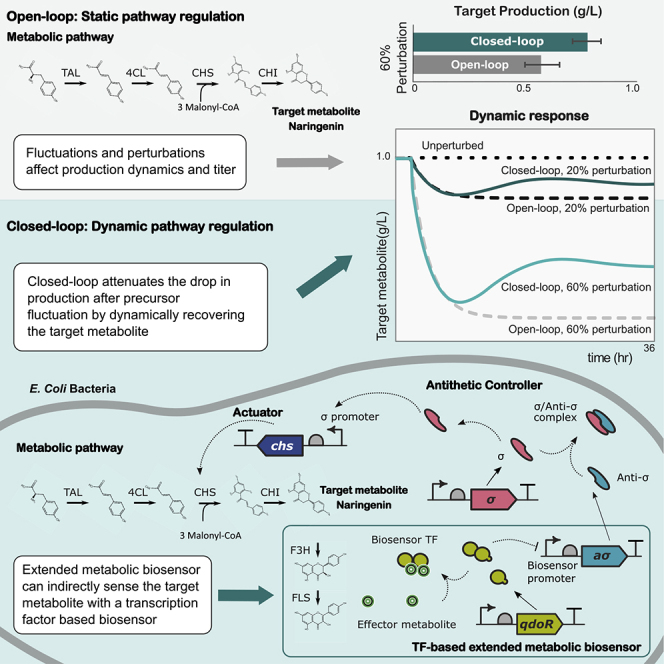

## Introduction

Natural cells maintain robust growth and withstand environmental fluctuations by dynamically adjusting cellular metabolism through complex regulatory networks (Liu et al., 2018). Since nature has optimized metabolite production for its needs, these specific optimal solutions are usually not compatible with industry-level overproduction demands. Major improvements in yield, titer, and productivity of engineered metabolic pathways can be accomplished by balancing pathway gene expression (Liu et al., 2018). The objective is to increase production of the target product through reducing potential flux imbalances in the host organism. This is mainly accomplished by eliminating the production of excessive intermediate metabolites and precursors leading to efficient conversion of intermediates, substrates, and co-factors to desired products. There exist several metabolic pathway-balancing approaches that optimize gene expression and flux distribution based on *in silico* predictions provided by static constraint-based metabolic genome-scale models (Purdy and Reed, 2017), using regulatory elements (DNA copy number, promoter and ribosome binding site [RBS] engineering) (Nielsen et al., 2016), synthetic scaffolds, compartmentalization, and flux diversion (silencing, knockouts, alternative carbon sources) (Chae et al., 2017). These pathway regulation strategies optimizing for a particular condition are static, so they are unable to respond to growth and environmental changes that occur in a bioreactor setup (Shi et al., 2018; Wehrs et al., 2019). Moreover, these static control systems may not be suitable when piecing together a complicated pathway with biosynthetic modules with mismatched input/output levels or when there is a need to minimize the accumulation of potentially toxic intermediates (Shi et al., 2018).

Dynamic balancing addresses the robustness pitfalls of static control through the application of feedback and feedforward regulation. This makes it possible to attain higher titers as compared with static regulation (Stevens and Carothers, 2015). However, it is not until recent years that metabolic engineers have used dynamic regulation to redirect endogenous flux toward product formation, balance the production and consumption rates of key intermediates, and suppress production of toxic intermediates in the fermentation (Doong et al., 2018). The main reason is that, in order to implement a dynamic regulation strategy, biosensors are needed. Indeed, a dynamic regulation system consists of a sensing component, which can detect the metabolite of interest or physiological state (e.g., growth, stress signals), and a regulator component, which converts the sensor signal into a transcriptional signal, often resulting in the upregulation or downregulation of a key pathway gene (Paepe et al., 2018; Huyett et al., 2018; Liu and Zhang, 2018).

Despite a growing number of success stories, engineering dynamic control remains extremely challenging (Chen and Liu, 2018; Gao et al., 2019). Among these challenges, one can count cell resource allocation, microorganism population heterogeneity, and fluctuating industry-scale bioreactor environment. Moreover, the performance specifications for synthetic gene circuits and components change significantly with variations in parameters such as temperature, host organism, growth media formulation, and position of the genes in the genome (Segall-Shapiro et al., 2018). To address these challenges, model-based design relying on the principles of control engineering can provide a powerful formalism to engineer dynamic control circuits. These, together with the tools of synthetic biology, can lead to robust and efficient microbial production at industrial levels (Liu et al., 2018; Segall-Shapiro et al., 2018; Hsiao et al., 2018; Shopera et al., 2017; Boada et al., 2017a, 2017b).

Biosensor mechanisms that translate information about a chemical signal (the concentration of a natural product) into a measurable output are being increasingly used in engineering and synthetic biology (Johnson et al., 2017; Shi et al., 2018; D'Ambrosio and Jensen, 2017). Intracellular biosensors can be broadly grouped into three categories, RNA switchers (aptamers) (McKeague et al., 2016), reporter-proteins leading to signal generation (Agrawal et al., 2020), and transcription factors (TF) leading to transcriptional regulation (Mahr and Frunzke, 2015), on the basis of their biomolecular make-up and mechanism. TF-based biosensors present advantages in terms of specificity and sensitivity (Lin et al., 2017). However, the use of biosensors in synthetic circuits to control gene expression is in its beginnings, as the tunability of the biosensor is essential for dynamic control systems to appropriately function in industrial conditions (Liu et al., 2018; Wang et al., 2019). Only recently, libraries of TF-based biosensors have been created by varying regulatory elements such as RBS (Paepe et al., 2018). Also, the biosensor dynamic range and thresholds can nowadays be modified through directed evolution (Snoek et al., 2019) or with the use of model-based design for the constraints of dose-response curves (Mannan et al., 2017). However, reported chemicals that can be detected by TF-based biosensors are generally focused on some specific classes of compounds such as amino acids and do not cover the entire range of nature's chemical diversity. SensiPath (Delépine et al., 2016) has been recently introduced to develop extended TF-based biosensors through metabolic pathways, thus expanding the observable chemical space spanned by biosensors.

Extended metabolic biosensor-based circuits considerably enlarge the ability of sensing target molecules for pathway screening and regulation. Such type of genetic circuits can be computationally designed through an *in silico* screening of the extended metabolic space (Carbonell et al., 2014; Delépine et al., 2016). As the number of characterized transcription factors that are responsive to effector molecules is constantly increasing, the number of possible ways of setting up a genetic circuit encoding an extended metabolic biosensor for the desired target increases as well. Currently, the number of known small-molecule chemical effectors for transcription factors is close to 750 (Koch et al., 2018) providing a large design space that can be explored in order to select the optimal biosensor circuit depending on the objectives. Given the set of reachable metabolites, i.e., chemicals that can be produced through enzymatic transformations, and the set of effector metabolites that can induce or modulate the response of a transcription factor, we define the set of biosensor circuits as all possible biosynthetic metabolic pathways that can convert the desired target into one of the metabolites within the effectors set.

In order to streamline the design and use of TF-based biosensors for dynamic regulation of metabolic pathways, in this study we aimed at introducing an extended metabolic biosensor-based antithetic control for regulating the heterologous pathway of the flavonoid naringenin production in the *Escherichia coli* host. We chose the naringenin pathway as our case study because of the availability of both direct and indirect TF-based biosensors for intermediates and derivatives involved in the production of this class of compounds of industrial biomanufacturing interest. Flavonoids are an important subclass of phenylpropanoids, a major family of plant natural products with applications as food supplements, antioxidants, aroma and flavoring agents, pharmacological drugs, insecticides, and dyes. Clear market opportunities exist for flavonoids with enhanced bioavailability and bioactivity profiles used as flavors and bioactive compounds for nutraceutical applications among others. The flavonoid compound naringenin is predominantly found in grapefruits and oranges and has been reported to have many pharmacological properties, including anti-dyslipidemic, anti-obesity and anti-diabetic, and anti-fibrotic (Liu et al., 2008; Rahigude et al., 2012; Zygmunt et al., 2010). It also has a central place in the biosynthesis of all flavonoids, as naringenin plays an important role as backbone scaffold that can be further derivatized. Downstream pathways may add biochemical groups on it. These molecular modifications (derivatizations) are responsible for functionalization and diversification of end products in the broad families of flavonoids.

Although microbial fermentation of naringenin has been achieved, current titers do not render its microbial production economically feasible. Current maximum reported titers for production of naringenin in industrial hosts are close to 200 mg L^−1^ (Zhou et al., 2019). However, industrial levels above 1 g L^−1^ might be possible to reach through further optimization toward the maximum theoretical yield. Notably, Juminaga et al. (2011) optimized the production of the L-tyrosine precursor in *E*. *coli*, achieving a yield of 0.44 g *L*-tyrosine/g glucose, which was determined to correspond to 80% of the maximum theoretical yield. The obtained yield allowed the production of 2.2 g L^−1^ of *L*-tyrosine in 48 h. Moreover, a titer of 474 mg L^−1^ of naringenin has been reported when feeding *p*-coumarate by host optimization (Xu et al., 2011). Therefore, we expect that the titers can go still up, especially because of the high efficiency of the conversion of *L*-tyrosine into *p*-coumarate, and should be able to reach at least 0.4 g naringenin/g glucose and a titer of 800 mg L^−1^ in 48 h in a fermenter, with still some room for further optimization through additional strain optimization, chromosome integration, and process optimization. Besides the increase in the amount of molecules per cell, the final titers of naringenin in *E. coli* can also be significantly increased by using high-density culture bioreactors, which can reach cell concentrations above 100 OD (Choi et al., 2006).

The naringenin pathway consists of four enzymatic steps from the *L*-tyrosine precursor. The third step, catalyzed by naringenin chalcone synthase (CHS) requires malonyl-CoA, an essential metabolite that is used in fatty acid production and plays an important role in cell metabolism. Intracellular concentrations of malonyl-CoA are typically low (4–40 μM in *E. coli*) (Johnson et al., 2017; Xu et al., 2014). Moreover, its concentration is subject to fluctuations caused by cell environmental heterogeneities. Several strategies have been used in order to channel the malonyl-CoA flux into the desired production pathway, including overexpression of the enzyme that synthetizes it or down-regulation (Yang et al., 2015). However, accumulation of malonyl-CoA leads to growth inhibition. Therefore, just-in-time dynamic production of malonyl-CoA appears as a desired goal.

Dynamic redistribution of cellular resources and optimal control of pathway expression offer alternative strategies for engineering metabolic pathways with high productivity and yield. In Xu et al. (2014) the FapR TF-based malonyl-CoA biosensor (Johnson et al., 2017) is used to design a metabolic switch that enables dynamic regulation of both the malonyl-CoA source pathway and its sink pathway in order to dynamically regulate malonyl-CoA concentration. The engineered strain improved fatty acids production as it reached a better trade-off between cell growth and heterologous pathway expression. As an alternative, Dinh et al. (2020) obtained a 60% titer increase in the production of naringenin by controlling the composition of a co-culture using a quorum sensing-based growth-regulation circuit.

**Figure 1:**
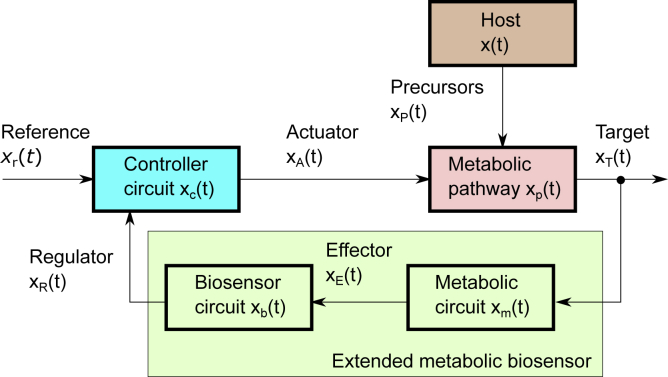
Schematic Diagram of the Proposed Strategy A target-producer metabolic pathway is expressed in the host, where the fluctuating availability of precursors *x*_*p*_(*t*) act as perturbations that eventually affect the target metabolite production *x*_*T*_(*t*). This is sensed using an extended metabolic biosensor device comprising a metabolic pathway that converts the target into an effector metabolite *x*_*E*_(*t*) and an associated transcription factor-based biosensor that provides the regulation signal *x*_*R*_(*t*). This one is fed back to a gene regulatory circuit that expresses the actuator signal *x*_*A*_(*t*) driving the target metabolite toward the specified set-point *x*_*r*_(*t*). In our case, the actuator signal *x*_*A*_(*t*) consists of a parallel expression system for the limiting precursor *x*_*p*_(*t*).

Here, we will use a different approach where a naringenin biosensor is used to establish a feedback control system that will regulate the levels of expression of the naringenin chalcone synthase enzyme in order to provide a robust response to the fluctuations in malonyl-CoA availability and maximize the production of the naringenin target. Notably, our strategy, as depicted in Figure 1, will consist of initially establishing a baseline production pathway for naringenin and to use an extended biosensor-based feedback control system to regulate naringenin production around its nominal level while coping with fluctuations in the malonyl-CoA availability.

## Results

### Response of the Naringenin TF-based Extended Metabolic Biosensor

The FdeR transcription factor is responsive to naringenin and has been used as a biosensor for high-throughput screening. However, its dynamic range is 0.001–0.15 mM, i.e., it saturates around 40 mg L^−1^, which means that, even if that biosensor could be used during the prototyping stage to implement pathway regulation, its use for feedback regulation is not viable at the desired industrial levels. Therefore, it is necessary to search for a transcription factor-based flavonoid biosensor, such as those reported in the literature (Cheng et al., 2018), that performs an indirect measurement allowing tuning of the dynamic range.

A promising case is a biosensor circuit based on the transcription factor QdoR for kaempferol and quercetin, two flavonols that can be derived from naringenin in two and three conversion steps, respectively. This transcription factor, with a similar dynamic range than the one for naringenin, can provide an extended metabolic biosensor-based solution for naringenin high-producer strains. Conversion ratios for quercetin of 13:1 in *E. coli* (Leonard et al., 2006) and of 100:3 in yeast (Trantas et al., 2009) have been reported; therefore, it is possible to extend the net dynamic range for naringenin above 1 g L^−1^ even if the use of a QdoR-based biosensor compared with a FdeR-based one would introduce some additional challenges. One challenge of this approach is that, in order to produce kaempferol or quercetin, naringenin needs to be consumed and some of the intermediates might accumulate at high concentrations. However, a recent study has shown that, through appropriate selection of the enzymes in the pathway, it is possible to tune the levels of accumulation of each intermediate (Rodriguez et al., 2017). In that study, production for each intermediate was optimized by trying to reduce accumulation of the other compounds. For the biosensor case, the goal would be to keep naringenin at high levels with a low conversion ratio to kaempferol, and a minimal accumulation of the intermediate dihydrokaempferol.

**Figure 2:**
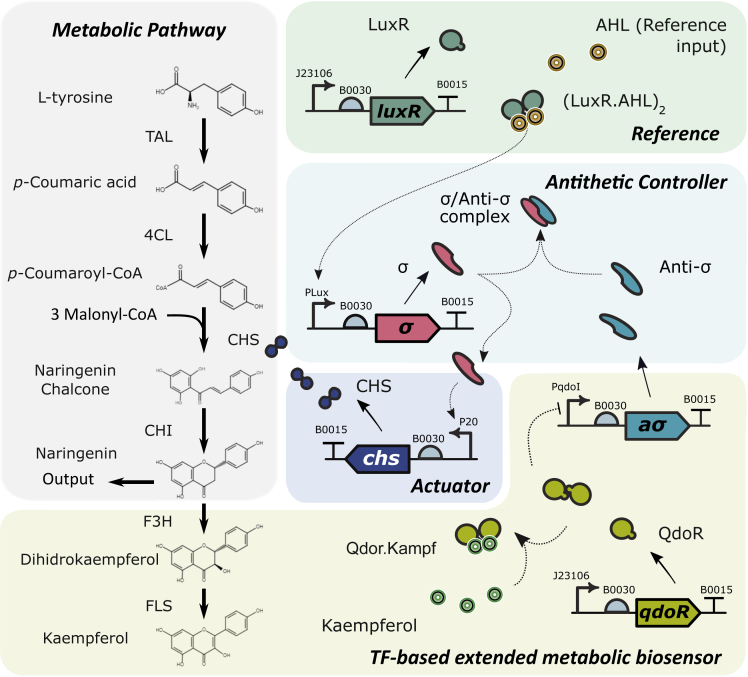
Naringenin Pathway, Antithetic Controller and TF-Based Extended Metabolic Biosensor Production of the naringenin target is proxied by a metabolic circuit through the downstream metabolite kaempferol, which is sensed by the QdoR transcription factor and feeds back to an antithetic controller. The controller is activated upstream by the external AHL inducer, and its actuating signal overdrives the expression of the CHS enzyme in the pathway, in order to compensate for malonyl-CoA depletion.

The TF-based extended metabolic biosensor proposed in this work, depicted in Figure 2, uses the downstream metabolite kaempferol as proxy of naringenin. Kaempferol in turn captures the QdoR transcription factor, which represses the expression of the anti-*σ* molecule by means of the qdoR-PqdoI promoter region (Siedler et al., 2014). In this way, increasing values of naringenin will produce increasing values of anti−*σ*. Thus, the naringenin TF-based extended biosensor starting from naringenin generates the sensor input signal, and the sensor output signal coming from anti−*σ* is fed back to the controller. The resulting extended biosensor was modeled as described in Transparent Methods section in the Supplemental Information.

**Figure 3:**
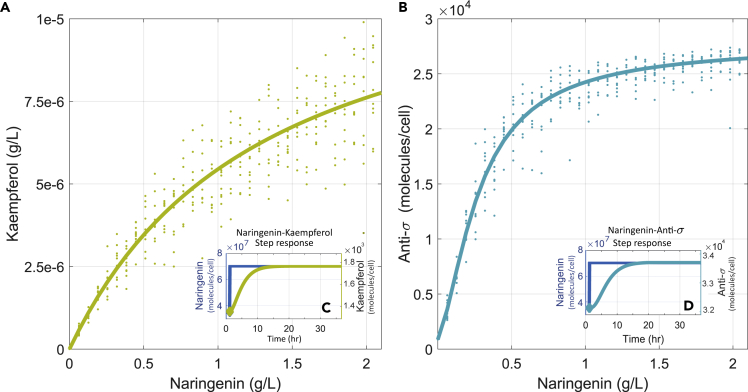
Naringenin TF-based Extended Metabolic Biosensor Dose-Response (A–D) (A) Naringenin to kaempferol dose-response showing the kaempferol concentration (g L^−1^) corresponding to a varying range of naringenin production concentrations (g L^−1^). The inset (C) shows the time response of kaempferol to a sudden step-like change in the concentration of naringenin concentration. (B) TF-based extended metabolic biosensor input-output dose-response. The amount of anti-σ molecules is shown for a varying range of naringenin production levels (g L^−1^). The inset (D) shows the time response of anti-σ to a sudden step-like change in the concentration of naringenin concentration. In both graphs solid lines are the mean values and dots correspond to the dose-responses under a 15% uncertainty in the biosensor parameters for a plasmid copy number of ten.

Figure 3 shows the dynamic range of the biosensor according to its dose-response curve. For instance, to sense 1 g L^−1^ of naringenin, the extended biosensor needs to convert only 5 μg L^−1^ of kaempferol. This means that the extended biosensor has a very low gain, allowing the biosensor promoter to work in the non-saturated region while responding to changes of naringenin concentration in the g L^−1^ range. To that end, the metabolic part of the extended biosensor had to be tuned in such a way that it produces very low amounts of kaempferol for large amounts of naringenin. This can be achieved, for instance, by tuning for a very low affinity to the substrate. This is particularly relevant for the first enzyme of the biosensor. In a similar fashion, the rest of the enzymes of the extended pathway need to show low efficiency. Based on such designated enzyme configuration, parameters in the system were adjusted as described in Metabolic pathway and Table S1 in the Supplemental Information, and the corresponding dynamic response of the biosensor was analyzed. The insets of Figure 3 show the time response of the naringenin extended metabolic biosensor. Figure 3C shows the time response of kaempferol to a sudden change in the naringenin concentration, whereas Figure 3D shows the time response of anti-σ, the sensor output signal. As expected, similar dynamics between a change in the naringenin concentration and the response to that change were found in both concentrations of kaempferol and anti-σ. The apparent delay introduced by the slow dynamics can be mainly attributed to the dilution of kaempferol due to cell growth. Interestingly, as detailed later, the integral antithetic controller is able to provide the desired robust response, despite this extra dynamics in the loop. Notice also that the low gain of the biosensor contributes to reduce the metabolic burden it introduces.

### Response of the Antithetic Integral Feedback Regulator

To adopt the use of extended metabolic biosensor circuits in pathway regulation, important effects may impact their performance; therefore, they need to be considered. Such effects appear because of the delays and nonlinearities introduced by the metabolic circuit. For screening applications, they can often be compensated through calibration of the biosensor. In pathway dynamic regulation nevertheless, the effects can have a strong impact on the performance of the pathway and even bring it to unstable behavior (Hsiao et al., 2018). Hence, it is necessary to establish a control strategy for counteracting the inherent uncertainties of the response for a given design and to determine the conditions and ranges that must be verified by the extended metabolic biosensor parameters in order to guarantee a stable and robust response.

To that end, integral feedback control appears as a promising solution for robust output regulation against perturbations. The antithetic feedback control is a type of integral control that has been recently shown to be a universal genetic topology that can achieve robust perfect adaptation (Briat et al., 2016) and has already been considered for regulation of metabolic pathways (Briat and Khammash, 2018). Therefore, the use of a genetic circuit implementing an antithetic integral feedback controller in combination with an extended metabolic biosensor seems a promising genetic system for pathway regulation.

**Figure 4:**
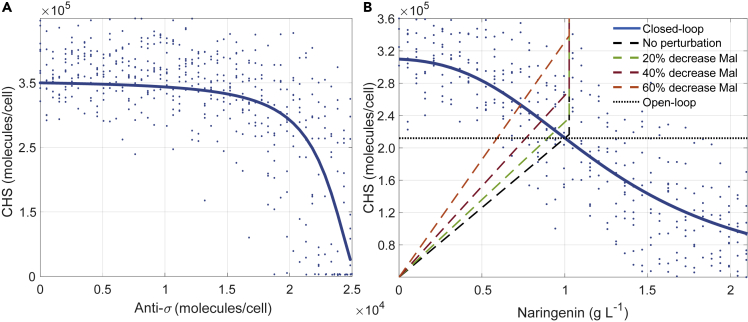
Antithetic Controller Input-Output Dose-Response (A) The amount of CHS molecules is shown for a varying range of the amount of anti-*σ* molecules produced by the TF-based extended metabolic biosensor. (B) Production curve and TF-based extended biosensor/controller combined dose-response. Closed (blue) and open loop (black dotted) dose-responses. Dashed lines represent the relationship between the production of naringenin and the amount of CHS enzyme for different amounts of malonyl-CoA (for fixed pathway conditions). In both graphs solid lines are the mean values and dots correspond to the dose-responses under a 15% uncertainty in the biosensor parameters for a plasmid copy number of ten.

Here we use the antithetic control structure, as depicted in Figure 2, to dynamically express the enzyme CHS catalyzing the conversion from *p*-coumaric acid to naringenin chalcone. The decrease in naringenin implies less amount of both kaempferol and anti−*σ* factor (Rhodius et al., 2013), and its σ cofactor will no longer be suppressed. Mutual annihilation of the antithetic σ/Anti-σ cofactors provide negative integral feedback (Aoki et al., 2019) to regulate CHS enzyme production under the *σ*−P20 promoter region. σ Cofactor is activated by the upstream protein LuxR together with the external inducer AHL. Figure 4A illustrates how the CHS enzyme expression is reduced when the σ/Anti-σ negative feedback acts (increasing expression of anti-σ).

Notice that the global amount of CHS in the cell directly modulates the conversion flux from *p*-coumaric acid to naringenin chalcone (see Metabolic pathway in the Supplemental Information). The antithetic controller dynamically provides a variable amount of CHS additional to its basal level of expression. This way the controller compensates for fluctuations affecting the production of naringenin. The dynamics of the controller was modeled as described in section Antithetic controller in the Supplemental Information.

Putting together the previous three dose-response curves one can obtain the dose-response curve (static relationship) of the combined TF-based extended biosensor controller (Figure 4B). This combined dose-response is the complete input-output relationship of the feedback representing how the level of CHS enzyme expression will change upon changes in the naringenin levels. Within the same Figure 4B we also represented in dashed colored lines the production curves that represent the relationship between the production of naringenin and the amount of CHS enzyme for different fixed amounts of malonyl-CoA (for fixed pathway conditions). Note that there is a threshold in the transition above 1 g L^−1^. As the production curves are calculated for fixed pathway conditions that were selected for nominal levels of 1 g L^−1^, for higher production values, the rest of the pathways turn to be the limiting process and prevents higher levels of naringenin from being produced.

Once the amount of enzyme is selected, for instance, the amount shown in dotted black line in Figure 4B (representing the open-loop strategy, see Figure S1 in the Supplemental Information for more details, i.e., a fixed amount of enzyme), the intersection of this line with the dashed lines representing the production gives the amount of naringenin produced with that amount of enzyme. Here we see that the open loop strategy results in a decrease of levels of production upon malonyl-CoA reduction. Similarly, the closed-loop equilibrium is the intersection of the closed-loop dose-response curve with the production lines. In that case, it is possible to see how the closed loop can recover from a reduction in malonyl-CoA availability thanks to the dynamical regulation of the amount of enzyme.

### Dynamic Regulation of the Naringenin Pathway

**Figure 5:**
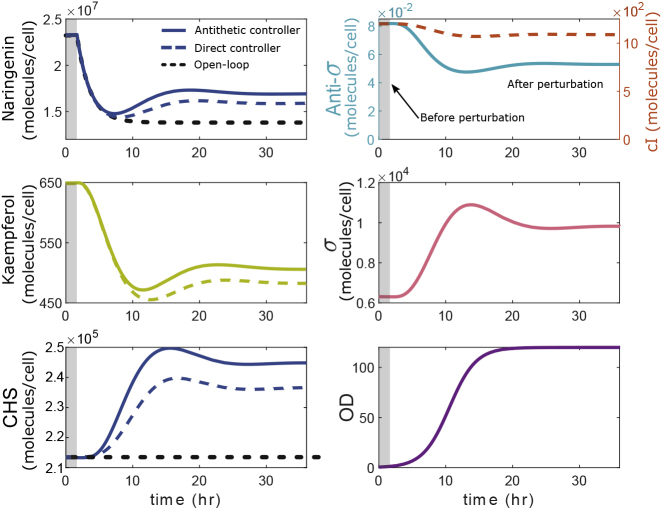
Dynamic Response of the Regulation of the Naringenin Pathway in the Presence of Perturbations Comparison among the dynamic responses of the antithetic controller (solid line), direct controller (colored dashed line), and open-loop (black dashed line) for a malonyl-CoA perturbations of 60%. Time course variation in naringenin, CHS, and cellular growth (OD) for the three cases. Time course variation of the kaempferol response for both controllers. Time course variations of anti-σ and σ for the antithetic controller and cI for the direct controller. Perturbation occurs at t = 1 h. The gray area corresponds to the response before the perturbation is applied, and white area afterward. See also Figure S1 and Figure S2.

In order to analyze the pathway dynamic regulation and eventually design a robust feedback regulation, the dynamic response of the two enzymatic steps in the extended biosensor in Figure 2 needs to be considered. The kinetics of the involved enzymes, however, have been less studied than of those in the naringenin pathway, and therefore, it would be necessary to assume some level of uncertainty in the parameters associated with the dynamic response of the biosensor. Here, we will assume typical kinetic values for the enzymes in the pathway extracted from the literature (see Methods). Figure 5 depicts the performance assessment of the regulated pathway assuming a model for the system as described in Methods. The output response in closed loop (Figure 5, solid lines), i.e., regulation based on the kaempferol-mediated naringenin biosensor driving an antithetic integral controller (see Figure 2) was compared with the output response in open loop (Figure 5, dashed black line), i.e., without feedback regulation of the CHS concentration (see Figure S1). Moreover, we also compared our strategy with a regulation based on a kaempferol-mediated naringenin biosensor driving a direct feedback controller (Figure 5, dashed blue line), i.e., a repressible promoter (cI regulated promoter) directly driving the expression of CHS enzyme (see Figure S2 and the direct controller model in the Supplemental Information).

The open-loop response results by replacing the P20 promoter that responds to σ (and in turn to naringenin via the biosensor) with a constitutive promoter (see Figure S1 and the open-loop model in the Supplemental Information for more details). This promoter was selected in such way that for the same conditions (same flux of L-tyrosine and malonyl-CoA) it provides the same amount of naringenin as the closed-loop circuit (around 1 g L^−1^). The direct feedback controller (see Figure S2) involves a repressible promoter (cI regulated promoter) directly driving the expression of the enzyme CHS. In turn, the repressor (cI) is expressed from the same PqdoI promoter of the biosensor. The values of the parameters of this direct controller (see Table S2) were selected to provide the same amount of naringenin as the closed-loop circuit (around 1 g L^−1^) for the same conditions (same flux of L-tyrosine and malonyl-CoA).

**Figure 6:**
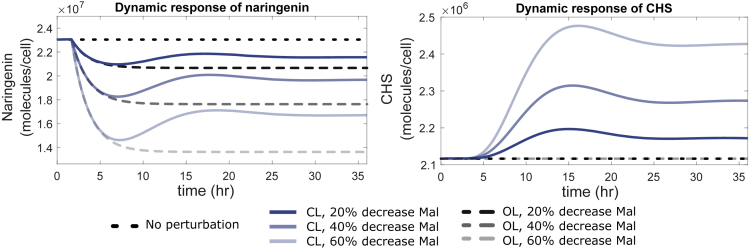
Comparison between Open-Loop and Closed-Loop Dynamic Responses for Malonyl-CoA Perturbations up to 60% (A) Output response for naringenin concentration for a decrease in malonyl-CoA availability of 20%, 40%, and 60% in open and closed-loop. (B) Time course variation in CHS concentration in the open-loop case (constant, no dynamic regulation) and the same changes in malonyl-CoA. See also Figure S1.

Figure 6 shows the transient responses for different values of malonyl-CoA decrease. The perturbation occurs at time *t* = 1 h. In the regulated case, the levels of production of naringenin were successfully recovered after a transient response of approximately 24–30 h, reaching the end of the experiment at 36 h with a steady production.

**Figure 7:**
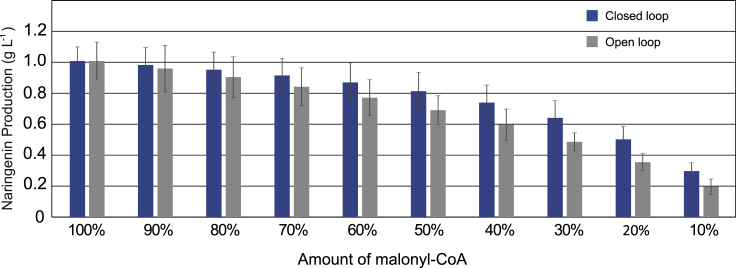
Naringenin Production Titers after 36 h under a Malonyl-CoA Perturbation in Open-loop Compared with the Closed-Loop Biosensor-Based Regulation Error bars represent standard deviations due to parameter uncertainty.

Figure 7 shows the predicted titers in naringenin production in open and closed loop obtained after 36 h. An initial titer close to 1 g L^−1^ of naringenin production is assumed in the engineered strain under optimal growth conditions. At time *t* = 1 h a perturbation occurs in the cell state leading to a decrease in the availability of the malonyl-CoA precursor. Such variability in malonyl-CoA intracellular concentration often occurs in environments where the cells are subjected to stress and environmental conditions are not uniform, as typically found in large fermenters. As expected, the depletion in a key metabolite critically impacted the naringenin pathway. For instance, a decrease of 40% in malonyl-CoA would lead to a decrease of about 25.0% in the concentration of naringenin. Such drop in production is, in turn, successfully attenuated by the closed-loop system, where the typical decrease in the naringenin titers is kept below 15.0%, a variation that is quite acceptable under industrial production conditions. Moreover, a 60% reduction in malonyl-CoA leads to a 40% drop in the production of naringenin, which can be attenuated up to around 25% in the closed-loop system. It is important to notice that these results were obtained for a set of parameters in the closed-loop system that were not optimized. Indeed, the results we obtained for the closed-loop regulation can be further improved by proper tuning of the controller and biosensor parameters. This is demonstrated by the fact that, in the case of malonyl-CoA decreasing down to a 60% of its nominal value, it is possible to keep the naringenin production over 96% of the nominal production for some values of parameters (see Supplemental Information).

### Robustness of the Antithetic Integral Feedback Regulator and Comparison with the Direct Controller

In order to analyze the robustness of the circuit, we performed a sensitivity analysis modifying the model parameters for both the antithetic controller and the direct one. The values of the parameters were varied using the ranges listed in Table S3 at the Supplemental Information, based on typical values found in the literature.

For the antithetic controller, of the 2,911 possible combinations of parameters, 776 resulted in a naringenin production around the nominal level, 1 ± 0.05 g L^−1^ (Figure S3). Meanwhile, 15,700 combinations resulted in a production between 0.4 and 0.9 g L^−1^, whereas the production levels for the remaining 565 combinations were found to be lower than 0.4 g L^−1^ (see Supplemental Information for further details on the Robustness analysis). In contrast, for the cI promoter direct controller, of the 2,193 combinations of parameters only 87 resulted in a naringenin production of 1 ± 0.05 g L^−1^. Meanwhile, 507 combinations resulted in a production between 0.4 and 0.9 g L^−1^ and the remaining 1,599 combinations in a production lower than 0.4 g L^−1^. Notice the parameters were varied between one and two orders of magnitude. In spite of such large variation, the antithetic controller successfully kept the output within the desired region of production in a region of parameters much broader than in the case of the direct controller. In addition, the antithetic controller demonstrates much less performance degradation, achieving a production over 0.4 g L^−1^ in more than 80% of the cases as compared with 27% cases with the direct controller.

What is found to be one of the most influential parameters of the biosensor and actuator was the plasmid copy number (Figure S4). To maintain the best performance (around 1 g L^−1^) we found that the plasmid copy number of the biosensor (C_Na*σ*_) must be 1 copy (250 solutions), 5 copies (230 solutions), or 10 copies (180 solutions). All together these three values represent more than 80% of the best solutions. With respect to the plasmid copy number of the actuator (C_Nh_) the results were 5 copies (407 solutions) and 10 copies (387 solutions). All together these two values represent 100% of the best solutions (see Supplemental Information for further details).

## Discussion

Creating robust and stable microbial strains that produce large amounts of the target chemical for a long period at fermenter cultivation is a desirable industrial goal. Successful implementations of this goal need to start by finding suitable designs during the prototyping stage. Establishing feedback regulation in the pathway brings better stability and reproducibility during the process scaling-up. To that end, the selection of an appropriate biosensor in the feedback loop is necessary. Some biosensor-based proposals require the direct measurement of the metabolite of interest (Liu et al., 2015) or are based on sensing growth (Dinh et al., 2020). However, an issue at industrial production is the difficulty of measuring the concentration of the target metabolite or signal of interest through a biosensor because of their typically limited dynamic range, adapted to those levels found in natural environments. Therefore, adjusting the biosensor parameters remains challenging and a strategy that relies on model-based design has been recently proposed by Mannan et al. (2017). Here, we introduce an approach that combines the indirect measurement of the chemical target with an integral antithetic feedback controller, which has been demonstrated robust against environmental variability (Briat et al., 2016). A circuit called extended metabolic biosensor is introduced where a small amount of the chemical target is converted through several enzymatic steps into an effector for some given transcription factor. In order to minimize biosensor consumption, our strategy consists of engineering a production pathway that provides the nominal levels and an additional closed-loop circuit intended to regulate small fluctuations in the baseline pathway due to changing environmental conditions often found during the scaling-up.

This approach not only opens up new venues to achieve robust pathway regulation but also involves several challenges. Notably, the indirect measurement performed by the extended metabolic biosensor creates some unavoidable delay in the feedback loop that can lead to instabilities. As shown in this study, a careful design of the circuit parameters is necessary in order to ensure stability and robustness in the response.

Another innovation in the proposed feedback regulation approach is that the control circuit is performed at a mid-point in the pathway, i.e., in the expression of the CHS enzyme to counterbalance the perturbation coming from the malonyl-CoA availability. We have shown here that identifying the weakest step or the bottleneck in the pathway is essential in order to design a feedback regulation-based strain. Other regulation points in the pathway were possible, but choosing the regulation of expression of the CHS enzyme was the best choice because it was directly involved in the response to the malonyl-CoA perturbation. Regulating the expression of the four-gene naringenin pathway instead of the CHS gene could have been an alternative. However, this strategy would have led to overshooting and bumped behavior because of the weak link between malonyl-CoA availability and some of the enzymes and reactions involved in the pathway.

A promising improvement based on investigating the link between the availability of malonyl-CoA and growth involves expanding the proposed regulation genetic circuit. Since the malonyl-CoA precursor is mainly consumed in the cell in order to produce fatty acids that eventually will feed the cell for growth, sensing malonyl-CoA could be considered as a proxy for cell growth as well as other cell phenotypic traits. Therefore, widely used malonyl-CoA TF-based biosensors can provide the possibility of improving the proposed regulation by coupling production to cell growth.

Our control strategy involves a continuous and low-proportion (10^6^:1) consumption of the target chemical through the extended metabolic biosensor in order to feed back the signal to the genetic controller. Tuning this metabolic pathway involves both reducing the affinity of the enzyme F3H to its substrate by a factor of 10^6^ and reducing the catalytic activity by a factor of 60. In fact, these modifications are not an issue, as they entail a reduction in the enzymatic activity (Arnold, 2018).

As shown in this study, the integration of metabolic circuits and TF-based biosensors in pathway regulation is a robust solution for the high-performance production of target chemicals in the engineered microbial strains that are currently designed in modern biofoundries (Carbonell et al., 2018). Our analysis of the dynamic response of a cell factory under an extended metabolic biosensor circuit and antithetic feedback control has shown promising robust results against external and parametric perturbations compared with a direct controller, allowing a more efficient experimental design. Such devices are expected to become increasingly embedded as standard parts plugged into engineered strains for chemical production, enabling rapid prototyping and robust scale-up of microbial production from microplate prototypes to industrial levels.

### Limitations of the Study

The present study analyzes an approach to synthetic pathway regulation based on indirect measurements and the use of an integral antithetic feedback control. In order to simulate the dynamic response of the system, we have focused on a study case for the production of the naringenin flavonoid and its enzyme dynamics has been approximated through Michaelis-Menten kinetics. Kinetic constants were obtained from enzyme databases based on *in vitro* assays reported in the literature in order to define the value ranges of the parameters. We might expect actual values to differ, and for that reason a range was defined rather than constant values.

The study has focused on the main intermediates in the pathway, ignoring other effects such as co-factor availability as well as environmental conditions. Therefore, the study provides a first approximation to the problem that focuses on intrinsic properties rather than external factors, which were lumped on the single effect of the malonyl-CoA as the main external perturbation. As accurate whole-cell models for *E. Coli* become available (Goldberg et al., 2018), studies on dynamic pathway regulation will become more detailed and will allow for a larger set of environmental conditions and cell states to be tested. Future work includes extending the model to incorporate the malonyl-CoA dynamics and its relationship with the central metabolism and nutrients availability in the bioreactor. However, our current model does not include the metabolism of the cell and does not consider the possible implications in terms of genetic or metabolic burden. It has been shown that incorporating a genetic circuit in the cell can lead to metabolic burden reducing the overall performance of the cell (Ceroni et al., 2018). In this respect, we also foresee as future work the integration of the proposed strategy with whole-cell models (Nikolados et al., 2019) taking into account for the metabolic burden that the circuit and pathway incorporation add to the system. This will allow us, along with an optimization-based tuning of the circuit parameters (Boada et al., 2019, 2016), to seek for the set of parameters yielding the best naringenin production while introducing the minimal metabolic burden.

Similarly, the present study did not take into consideration challenges such as stability of the genetic circuit when expressed through a plasmid vector rather than through genome integration, especially in fermentation processes where the strains are under mechanical and chemical stress and fluctuations in the environmental conditions (Wehrs et al., 2019; Hicks et al., 2019). Inhibition effects of high producer strains might also be taken into account when analyzing the robustness of the circuit. The present study did not take into account stochastic fluctuations in enzyme abundance. However, it is becoming increasingly clear that expression variation may propagate to metabolites (Evans et al., 2020) leading to a negative impact production. It is expected that, in a stochastic scenario, our strategy including the antithetic controller would have a good performance (Briat et al., 2016). Finally, another limitation that challenges the application of the proposed approach is that it involves an invasive biosensor and therefore it is necessary to tune the circuit not only in order to obtain an appropriate dynamic range and response but also in order to keep a suitable conversion ratio of the product.

### Resource Availability

#### Lead Contact

Further information and requests for resources should be directed to and will be fulfilled by the Lead Contact, Pablo Carbonell (pjcarbon@isa.upv.es).

#### Materials Availability

This study did not generate new unique reagents.

#### Data and Code Availability

Code for the models and simulations is available through GitHub (https://github.com/sb2cl/EMBA).

All the data obtained from the implemented mathematical model has been published as a Mendeley dataset (https://doi.org/10.17632/hpxhkyvctb.2).

## Methods

All methods can be found in the accompanying Transparent Methods supplemental file.

## Supplemental Information

Document S1. Transparent Methods, Figures S1–S4, and Tables S1–S3

## Footnotes

Supplemental Information can be found online at https://doi.org/10.1016/j.isci.2020.101305.
