# Supplementary material for: Extended Metabolic Biosensor Design for Dynamic Pathway Regulation of Cell Factories
Source: iScience. 2020 Jun 23;23(7):101305. doi: 10.1016/j.isci.2020.101305 (PMC7334618; doi:10.1016/j.isci.2020.101305)
Supplement: Document S1. Transparent Methods, Figures S1–S4, and Tables S1–S3 [file mmc1.pdf]

iScience, Volume *23*

Supplemental Information

Extended Metabolic Biosensor Design

for Dynamic Pathway Regulation of Cell Factories

Yadira Boada, Alejandro Vignoni, Jesús Picó, and Pablo Carbonell

Supplemental Information

Extended metabolic biosensor design for dynamic pathway regulation of cell factories

1. Transparent Methods

1.1. Metabolic pathway

For every $i-$cell, the kinetics of the enzyme-catalyzed reactions involved in the metabolic pathway from L-tyrosine to naringenin (see Figure 2 in the main text) were modeled as the set of rate equations (1) obtained from mass balance equations and considering dilution due to cell growth rate μ.

$$
\begin{aligned}
\frac{d[Lt]}{dt} &= V_0 - V_{Lt} - \mu[Lt] \\
\frac{d[pC]}{dt} &= V_{Lt} - V_{pC} - \mu[pC] \\
\frac{d[pA]}{dt} &= V_{pC} - V_{pA,Ma} - \mu[pA] \\
\frac{d[Nc]}{dt} &= V_{pA,Ma} - V_{Nc} - \mu[Nc] \\
\frac{d[N]}{dt} &= V_{Nc} - V_{N} - \mu[N]
\end{aligned} \tag{1}
$$

For each reaction, V_j are the fluxes (molecules·min^{-1}). Lt is the number of molecules of L-tyrosine, pC is p-coumaric acid, pA is p-coumaroyl-CoA, Nc is naringenin chalcone, and N is naringenin, the flavonoid of interest. Ma is the number of molecules of malonyl-CoA, which is considered as a perturbation signal in the system.

We assume the flux V_0 corresponding to the precursor L-tyrosine keeps constant (see Table S1), and all fluxes V_j obey Michaelis-Menten kinetics (Michaelis and Menten, 1913) as follows:

$$
\begin{aligned}
V_0 &= K_{Lt} \\
V_{Lt} &= \mathrm{k_{catTAL}}[TAL]\frac{[Lt]}{\mathrm{K_{mLt}} + [Lt]} \\
V_{pC} &= \mathrm{k_{cat4CL}}[4CL]\frac{[pC]}{\mathrm{K_{mpC}} + [pC]} \\
V_{pA} &= \mathrm{k_{catCHS}}[CHS]\frac{[pA][Ma]}{\mathrm{K_{mpA}}\,\mathrm{K_{mMa}} + \mathrm{K_{mMa}}[pA] + \mathrm{K_{mpA}}[Ma] + [pA][Ma]} \\
V_{Nc} &= \mathrm{k_{catCHI}}[CHI]\frac{[Nc]}{\mathrm{K_{mNc}} + [Nc]} \\
V_{N} &= \mathrm{k_{catF3H}}[F3H]\frac{[N]}{\mathrm{K_{mN}} + [N]}
\end{aligned}
$$

where $\mathrm{k_{catj}}$ is the catalytic rate of each enzyme (min^{-1}), and K_{mj} the Michaelis-Menten constant for each substrate. The enzyme kinetic parameters, detailed in Table S1, were obtained from Brenda (Schomburg et al., 2017).

Metabolic pathway			
**Parameter**	**Description**	**Value**	**Unit**
TAL	Enzyme amount	3.2e5	molec
4CL	Enzyme amount	6.48e5	molec
CHI	Enzyme amount	3.54e5	molec
F3H	Enzyme amount	2.81	molec
FLS	Enzyme amount	5.84	molec
CHS	Basal (open loop) enzyme amount	2.13e5	molec
K_{Lt}	L-tyrosine flux	2e6	molec · min^{-1}
M	Malonyl-CoA initial amount	2.34e4	molec
k_{catTAL}	TAL catalytic constant	1.2	min^{-1}
k_{cat4CL}	4CL catalytic constant	0.492	min^{-1}
k_{catCHS}	CHS catalytic constant	1.68	min^{-1}
k_{catCHI}	CHI catalytic constant	4.2	min^{-1}
k_{catF3H}	F3H catalytic constant	174	min^{-1}
k_{catFLS}	FLS catalytic constant	6	min^{-1}
K_{mLt}	Michaelis constant $TAL - Lt$	1.9e4	molec
K_{mpC}	Michaelis constant $4CL - pC$	1.4e4	molec
K_a	constant $CHS - M$	1e-3	molec
K_b	constant $CHS - pC$	1e-3	molec
K_{mNc}	Michaelis constant $CHI - Nc$	2.8e4	molec
K_{mN}	Michaelis constant $F3H - N$	5e8	molec
K_{mDi}	Michaelis constant $FHS - Di$	1e4	molec

Table S1: Parameters for the metabolic pathway from L-tyrosine to kaempferol (Schomburg et al., 2017). Related to Figures 3 and 5.

The amounts of the enzymes TAL, 4CL, CHI and CHS corresponding to the pathway between L-tyrosine and naringenin were chosen so that the flux of precursor L-tyrosine can yield the targeted 1 g L^{-1} of naringenin (see Table S2).

Intracellular malonyl-CoA concentration is usually tightly regulated and maintained at very low levels in the cell (Yang et al., 2015). Therefore, for efficient production of malonyl-CoA derived molecules, enrichment of the intracellular malonyl-CoA pool is the standard practice. We considered a basal value of malonyl-CoA in the mid range of values reported in the literature (Wu et al., 2015; Takamura and Nomura, 1988; Xu et al., 2014). We avoid accumulation of large amounts of malonyl-CoA that may lead to growth inhibition.

1.2. ***Feedback regulation using the extended metabolic TF-based biosensor and the antithetic controller***

Next we model the feedback loop comprising the extended metabolic TF-based biosensor and the antithetic controller driving the co-expression of the enzyme CHS. Recall CHS is dynamically co-expressed through this feedback path in addition to its basal constant co-expression.

In this study, we focus on metabolic circuits that connect a target chemical to a transcription factor (TF)-based biosensor through their associated metabolic reactions. TF-based biosensors can be integrated as a genetic device into advanced sensing systems, defined here as TF-based extended metabolic biosensors, which consists of a proxy biosensor that detects changes in some target molecule by first transforming the molecule through one or several enzymatic steps into the effector molecule that drives the TF-based biosensor. Note that the target not necessarily has to be a single metabolite but is defined by a set of chemicals, which can correspond for instance to biomarkers or can be a proxy for biomass growth or other traits related to the strain phenotype and state. The extended biosensor space for a given target can be algorithmically mined through a metabolic expansion based on reaction rules (Delépine et al., 2016; Duigou et al., 2019). For an engineered production pathway involving n steps, each of the intermediates can be considered as targets for biosensors as well. Generally such intermediates are heterologous metabolites that do not interfere with other pathways in the host and therefore their detection through a biosensor can be used to probe different points in the engineered pathway.

Modeling TF-based extended biosensors requires the combination of the metabolic circuit transforming the target molecule T to the effector E and the TF-based biosensor. The dynamics of the circuit can be

approximated as the interconnection of two independent modules accounting for the metabolic circuit and the TF-based biosensor.

The metabolic circuit is typically modeled either using law mass action if concentrations of the enzymes are considered constant, or through Michaelis-Menten kinetics. We assume that gene expression of both the metabolic circuit and the biosensor is controlled by a single promoter (inducible or non-inducible).

The TF-based extended biosensor proposed in this work (Figure 2 in the manuscript) was modeled as follows.

1.2.1. Biosensor metabolic pathway stage

For every $i-$cell, and using the same assumptions as for the metabolic pathway between L-tyrosine and naringenin, the kinetics of the enzyme-catalyzed reactions involved in the metabolic pathway from naringenin to kaempferol (see Figure 2) were modeled as the set of rate equations (2):

$$\begin{aligned} \frac{d[Di]}{dt} &= V_N - V_{Di} - \mu[Di] \\ \frac{d[Ka]}{dt} &= V_{Di} - \mu[Ka] \end{aligned} \tag{2}$$

where V_j are the fluxes of each reaction (molecules·min^{-1}), Di is Dihydrokaempferol, and Ka is kaempferol, the effector flavonoid measured by the biosensor promoter. As for the pathway model (1), the flux V_{Di} obeys Michaelis-Menten kinetics:

$$V_{Di} = \mathrm{k_{catFLS}}[FLS]\frac{[Di]}{\mathrm{K_{mDi}} + [Di]}$$

The kinetic parameters, detailed in Table S1, were also obtained from Brenda (Schomburg et al., 2017). For the case of the enzymes involved in the extended metabolic biosensor we selected parameters from similar enzymes (eg. same product and different substrate). From the initial parameter range we tuned the values to achieve the desired gain for the biosensor. Directed evolution and statistical modeling can be used in order to experimentally tune the affinity and kinetics of the corresponding enzymes (Arnold, 2018; Berepiki et al., 2020).

1.2.2. Biosensor TF-based stage

Models describing the response of TF-based biosensors to changes in the effector concentration have been focused on determining the parameters of the dose-response curve, generally through a Hill function (Mannan et al., 2017; Trabelsi et al., 2018; Rogers et al., 2015, 2016). Performing a dose-response curve fitting is useful in order to determine static properties of the biosensor such as sensitivity, specificity or dynamic range, which are relevant for screening applications. However, employing a biosensor as part of a feedback circuit requires characterizing its dynamic response. Modeling the dynamics of TF-based biosensors has been less studied, with models either proposing apparent binding inhibition rate constants (with or without Hill's cooperativity) or data-driven models fitted to a linear system with delay.

Approaches to modeling the biosensor dynamics involve characterizing the time-dependent relationship between the input concentrations of the effector molecule and the target gene of interest through the dynamics of the biosensor internal states. Several dynamic models have been proposed in the literature. For instance, Zhang et al. (2012) developed a fatty acid/acyl-CoA biosensor based on the naturally occurring fatty acid-sensing protein FadR and its cognate regulator. The model for the biosensor in this study uses apparent rate constants for association and dissociation for FadR-ligand binding, as well as for association and dissociation of FadR-promoter complex. Gene expression, DNA replication and cell growth/species dilution are given by pseudo-first order rate constants. In another study, Feher et al. (2015) modeled the dynamic response of the malonyl-CoA biosensor considering production and dilution of the effector molecule and TF-binding to malonyl-CoA and promoter. The dynamic response was approximated to a second order linear system with a delay.

In our case, the TF-based stage of the biosensor uses the QdoR transcription factor, which represses the expression of the anti$-\sigma$ molecule by means of the qdoR-PqdoI promoter region (Siedler et al., 2014). The constitutive promoter J23106 (Anderson, accessed April 22, 2020) is used to express the QdoR transcription factor. Kaempferol captures the QdoR transcription factor, inactivating it. Equation (3) shows the resulting model for the dynamics of QdoR and anti$-\sigma$ as a function of QdoR and kaempferol.

$$\begin{aligned}\frac{d[Q]^i}{dt} &= \frac{\mathrm{p_Q C_N k_Q}}{\mathrm{dm_Q} + \mu} - (\mathrm{d_Q} + \mu)\,[Q] \\ \frac{d[a\sigma]^i}{dt} &= \frac{\mathrm{p}_{\mathrm{a}\sigma}\mathrm{C}_{\mathrm{N}_{\mathrm{a}\sigma}}\mathrm{k}_{\mathrm{a}\sigma}}{\mathrm{dm}_{\mathrm{a}\sigma} + \mu}\left(\alpha + \frac{(1-\alpha)\left(\mathrm{k_{dq}C_N}\right)^2\left(\mathrm{k_{dk}} + [Ka]\right)^2}{\left(\mathrm{k_{dq}C_N}\right)^2\left(\mathrm{k_{dk}} + [Ka]\right)^2 + \left(\mathrm{k_{dk}}[Q]\right)^2}\right) - \frac{\mathrm{k_{-c}}}{\mathrm{k_{dc}}}[\sigma][a\sigma] \\ &+ \mathrm{k_{-c}}[\sigma \cdot a\sigma] - (\mathrm{d}_{\mathrm{a}\sigma} + \mu)\,[a\sigma] \end{aligned} \tag{3}$$

where $a\sigma$ is the antifactor of σ protein, $\sigma \cdot a\sigma$ is the complex generated after σ sequestration (see section 1.2.3 below), Q is QdoR, and Ka is the amount of free kaempferol. All the parameters are listed in Table S1 and Table S2 .

To obtain the model (3), we considered the following assumptions for every i-cell:

- Transcription is fast enough as compared to translation, so it was assumed to be at quasi-steady state. Therefore, only the dynamics of proteins are considered.
- Translation is not a simple process (Alberts et al., 2009). It was modeled as an irreversible reaction with an average translation rate accounting for the fact that binding of ribosomes to the ribosome binding site (RBS) is indeed reversible, and several ribosomes may translate a single messenger RNA copy simultaneously.
- Degradation and dilution due to cells growth are considered for all species, including mRNA, with corresponding degradation rates $\mathrm{d_j}$ and specific growth rate μ.
- For each promoter, transcription activation or repression mediated by a transcription factor is modelled using a Hill-like function with the modification proposed in Trabelsi et al. (2018). This one accounts for the fact that the number of TFs and binding sites scale with the plasmid copy number.

1.2.3. Antithetic controller

Models based on ordinary differential equations for the antithetic feedback integral controller have been proposed in (Olsman et al., 2019; Aoki et al., 2019). In essence, the antithetic motif relies on the key mechanism of annihilation between the σ and anti$-\sigma$ factor and cofactor proteins. In our case, we considered the fact that the annihilation reaction, even though having a small dissociation constant, is reversible. As shown in Figure 2 from the manuscript, we considered production of the σ factor is induced by means of the dimer $(\mathrm{LuxR.AHL})_2$ using the PLux promoter. This way, the externally added amount of AHL acts as desired set-point for naringenin. Notice we do not assume the number of molecules of AHL needed to set a desired value for the ones of naringenin must be equal to this one – implying an unnecessary metabolic burden – but simply proportional. The free σ factor is a transcription factor for the promoter P20 used to express the naringenin chalcone synthase CHS, the output signal of the controller.

We considered the same assumptions as those used to derive the TF-based biosensor dynamics. The resulting model in equation (4) describes the dynamics of σ factor, the annihilation between σ and anti$-\sigma$,

and the production of CHS.

$$
\begin{aligned}
\frac{d[\sigma]^i}{dt} &= \frac{\mathrm{p}_\sigma \mathrm{C_N} \mathrm{k}_\sigma}{\mathrm{dm}_\sigma + \mu} \left(\alpha + \frac{(1-\alpha)[A]^2}{\mathrm{k_{dlux}} \left(\frac{\mathrm{k_{d2}C_N}}{[R]} \right)^2 + [A]^2} \right) - \frac{\mathrm{k_{-c}}}{\mathrm{k_{dc}}}[\sigma][a\sigma] + \mathrm{k_{-c}}[\sigma \cdot a\sigma] - (\mathrm{d}_\sigma + \mu)[\sigma] \\
\frac{d[\sigma \cdot a\sigma]^i}{dt} &= \frac{\mathrm{k_{-c}}}{\mathrm{k_{dc}}}[\sigma][a\sigma] - \mathrm{k_{-c}}[\sigma \cdot a\sigma] - (\mathrm{d_c} + \mu)[\sigma \cdot a\sigma] \\
\frac{d[CHS]^i}{dt} &= \beta \frac{\mathrm{p_{H_c} C_N k_H}}{\mathrm{dm_H} + \mu} + \frac{\mathrm{p_H C_N k_H}}{\mathrm{dm_H} + \mu} \left(\alpha + \frac{(1-\alpha)[\sigma]^2}{\mathrm{k_{d20}} (\mathrm{k_{d\sigma} C_N})^2 + [\sigma]^2} \right) - (\mathrm{d_H} + \mu)[CHS]
\end{aligned}
\tag{4}
$$

where A is the intracellular amount of AHL molecules (see below). All the parameters are listed in Table S2.

As mentioned above, we considered the desired set-point for naringenin is regulated by external addition of AHL, though indeed other extra- or intracellular signals could be used. In our case, we took into account that the interaction between AHL and AHL_e represent the physical passive diffusion process for cell-to-cell communication via quorum sensing. This was modelled as a reversible pseudo-reaction using mass-action kinetics, resulting in the dynamics given by equation (5)

$$
\begin{aligned}
\frac{d[R]^i}{dt} &= \frac{\mathrm{p_R C_N k_R}}{\mathrm{dm_R} + \mu} - (\mathrm{d_R} + \mu)[R] \\
\frac{d[A]^i}{dt} &= \mathrm{D}(\mathrm{V_c}[A_e] - [A]) - (\mathrm{d_A} + \mu)[A] \\
\frac{d[A_e]}{dt} &= \mathrm{D}\left(-x\mathrm{V_c}[A_e] + \sum_{i=1}^{x} [A] \right) - \mathrm{d_{Ae}}[A_e] \\
\frac{dx}{dt} &= \mu \left(1 - \frac{x}{\mathrm{x_{max}}} \right) x
\end{aligned}
\tag{5}
$$

where we also accounted for the dynamics of cell growth. R is LuxR protein; A and A_e are the intra and extracellular AHL molecules, respectively; and x is the number of cells in the culture. The term $\mathrm{V_c} = \frac{\mathrm{V_{cell}}}{\mathrm{V_{ext}}}$ is the ratio between the cellular and the environment volumes. As before, all the parameters are listed in Table S2.

1.3. *Static regulation in open-loop*

In the open-loop production of naringenin, the enzyme CHS is expressed constitutively as shown in the schematic in Figure S1.

For the $i-$cell, the same assumptions described in Methods section *Biosensor TF-based stage* and *Antithetic controller* from the manuscript were taken to design the direct controller model. Equation (6) describes expression of CHS enzyme.

$$
\frac{d[CHS]^i}{dt} = \frac{\mathrm{p_{H_c} C_N k_H}}{\mathrm{dm_H} + \mu} - (\mathrm{d_H} + \mu)[CHS] \tag{6}
$$

Parameter $\mathrm{p}_{H_c} = 9.8239\,\mathrm{min}^{-1}$ was chosen to give the same level of expression than in the closed-loop configuration for a fair comparison. All the other parameters are the ones listed in Table S2.

1.4. *Feedback regulation using a direct controller*

In the direct controller, cI protein replaces to σ and anti-σ cofactors from the antithetic controller as in Figure S2. The CHS enzyme is inhibited by the $lambda-$cI promoter region, when the TF-based biosensor detects an excess of naringenin and the subsequent kaempferol. This way, direct negative feedback regulates CHS expression, although reaching naringenin levels up to the 1 g L-1 set point is not guaranteed.

Biosensor and Antithetic controller gene circuit			
**Parameter**	**Description**	**Value**	**Unit**
μ	specific growth rate	$8.5e{-3}$	min^{-1}
α	tight basal expression	0.01	adim
β	constitutive expression	1.5	adim
$\mathrm{C_N}$, $\mathrm{C_{Na\sigma}}$	Plasmid copy number	10	copies
D	AHL diffusion rate across cell membrane	2	min^{-1}
$\mathrm{k_\sigma}$, $\mathrm{k_a\sigma}$	transcription rate	1.98	min^{-1}
$\mathrm{k_R}$	transcription rate	0.78	min^{-1}
$\mathrm{k_H}$	transcription rate	3.67	min^{-1}
$\mathrm{k_Q}$	transcription rate	0.71	min^{-1}
$\mathrm{p_\sigma}$	translation rate	3	min^{-1}
$\mathrm{p_{a\sigma}}$	translation rate	3.17	min^{-1}
$\mathrm{p_R}$	translation rate	2.34	min^{-1}
$\mathrm{p_H}$	translation rate	92.4	min^{-1}
$\mathrm{p_{Hc}}$	constitutive translation rate	$6.5e{-4}$	min^{-1}
$\mathrm{p_Q}$	translation rate	2.55	min^{-1}
$\mathrm{d_R}$	protein degradation rate	0.02	min^{-1}
$\mathrm{d_c}$	degradation rate $[\sigma \cdot a\sigma]$	$1e{-3}$	min^{-1}
$\mathrm{d_A}$	Intracellular degradation rate	$4e{-4}$	min^{-1}
$\mathrm{d_{Ae}}$	Extra cellular degradation rate	$4.8e{-5}$	min^{-1}
$\mathrm{kd_{20}}$	dissociation constant to $\mathrm{p_{20}}$	1000	molec
$\mathrm{kd_{lux}}$	dissociation constant to p_{lux}	600	molec
$\mathrm{kd_\sigma}$	dissociation constant σ dimer	1000	molec
$\mathrm{kd_q}$	dissociation constant to p_{qdoI}	150	molec
$\mathrm{kd_k}$	dissociation constant QdoR to kaempferol	75	molec
$\mathrm{kd_c}$	dissociation constant $[\sigma \cdot a\sigma]$	0.01	molec
$\mathrm{kd_{-c}}$	dissociation rate $[\sigma \cdot a\sigma]$	0.018	min^{-1}
$\mathrm{k_c}$	association rate $(\sigma \cdot a\sigma)$	1.8	min^{-1}
$\mathrm{dm_\sigma}$, $\mathrm{dm_{a\sigma}}$, $\mathrm{dm_R}$, $\mathrm{dm_H}$, $\mathrm{dm_Q}$	mRNA degradation rate	0.231	min^{-1}
$\mathrm{d_\sigma}$, $\mathrm{d_{a\sigma}}$, $\mathrm{d_H}$, $\mathrm{d_Q}$	protein degradation rate	$3e{-4}$	min^{-1}
**Comparison with cI direct controller gene circuit**			
$\mathrm{kd_{\lambda cI}}$	dissociation constant to $\mathrm{p_{\lambda cI}}$	1000	molec
$\mathrm{C_{N_{cI}}}$	Plasmid copy number	10	copies
$\mathrm{p_{hcI}}$	translation rate	54.89	min^{-1}
$\mathrm{p_{cI}}$	translation rate	3.17	min^{-1}

Table S2: Gene circuit parameters were taken from (Boada et al., 2017; Annunziata et al., 2017) and (Siedler et al., 2014). Related to Figures 3 and 5.

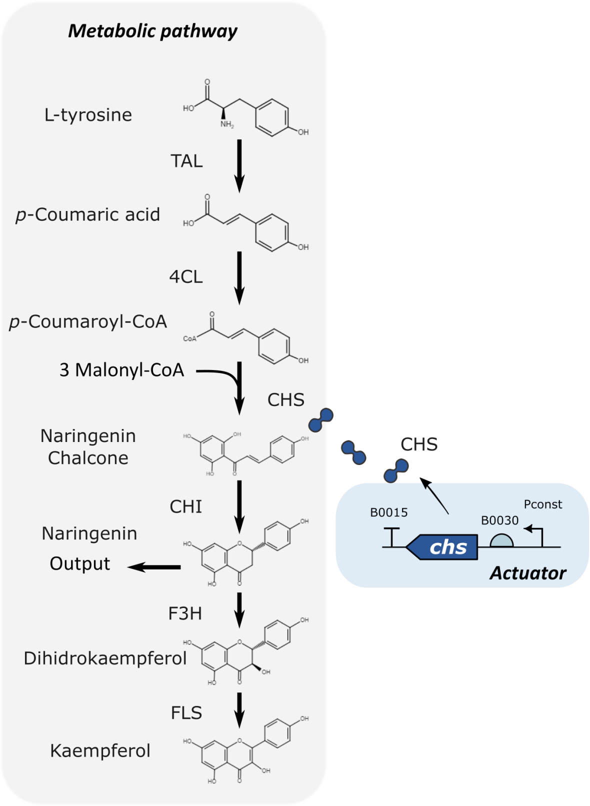

Figure S1: Open-loop configuration. Schematic of the naringerin biosynthesis pathway with constitutive CHS enzyme expression. Related to Figures 5 and 6.

For the $i-$cell, the same assumptions described in sections *Biosensor TF-based stage* and *Antithetic controller* from the manuscript were taken to design the direct controller model. Equation (7) describes the cI protein expression when the naringenin and kaempferol amount increased. Then, cI will repress the CHS production as in equation (8) until naringenin chalcone decreases. cI has a similar temporal dynamics of anti-σ.

$$\frac{d[cI]^i}{dt} = \frac{\mathrm{p_{cI}C_{N_{cI}}k_{cI}}}{\mathrm{dm_{cI}} + \mu}\left(\alpha + \frac{(1-\alpha)\left(\mathrm{k_{dq}C_N}\right)^2\left(\mathrm{k_{dk}} + [Ka]\right)^2}{\left(\mathrm{k_{dq}C_N}\right)^2\left(\mathrm{k_{dk}} + [Ka]\right)^2 + \left(\mathrm{k_{dk}}[Q]\right)^2}\right) - \left(\mathrm{d_{cI}} + \mu\right)[cI] \tag{7}$$

$$\frac{d[CHS]^i}{dt} = \frac{\mathrm{p_{H_c}C_Nk_H}}{\mathrm{dm_H} + \mu} + \frac{\mathrm{p_HC_Nk_H}}{\mathrm{dm_H} + \mu}\left(\alpha + \frac{(1-\alpha)[cI]^2}{\mathrm{k_{d20}}\left(\mathrm{k_{dcI}C_N}\right)^2 + [cI]^2}\right) - \left(\mathrm{d_H} + \mu\right)[CHS] \tag{8}$$

where cI is the cI protein, Q is QdoR, and Ka is the amount of free kaempferol. All the parameters are listed in Table 2 from the main text. Equations for QdoR production and for the naringerin metabolic pathway remain the same.

1.5. ***Robustness analysis***

In order to study how the different strategies respond to a variation on the values of the parameters, we selected the following parameters and ranges enumerated in Table S3.

For each parameter combination we obtain the naringenin production level without perturbation and with a 60% reduction in the available amount of malonyl-CoA. We performed this analysis for both the

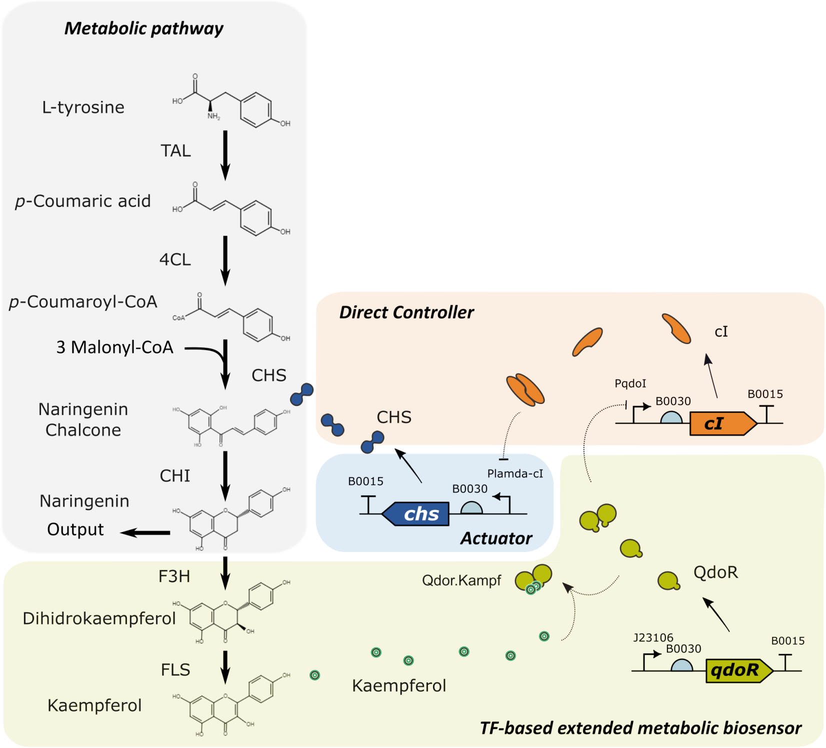

Figure S2: Direct feedback controller. Schematic of the naringenin biosynthesis pathway, where cI protein regulates CHS enzyme production. Related to Figure 5.

Robustness analysis			
**Parameter**	**Range**	**Nominal Value**	**Unit**
kd_{20}, $\mathrm{kd}_{\mathrm{lamcI}}$	$[250, 5500]$	1000	molec
kd_{q}	$[1, 500]$	150	molec
$\mathrm{CN}_{\mathrm{a}\sigma}$, $\mathrm{CN}_{\mathrm{cI}}$	$[1, 100]$	10	copies
$\mathrm{CN}_{\mathrm{CHS}}$	$[1, 100]$	10	copies
$\mathrm{p}_{\mathrm{a}\sigma}$, p_{cI}	$[0.5, 10]$	3.17	min^{-1}
p_{h}	$[1, 20]$	15.62	min^{-1}
$\mathrm{p}_{\mathrm{hcI}}$	$[3.5, 70]$	54.3	min^{-1}

Table S3: Range for the selected parameters. Related to Figure 2.

antithetic controller and the direct controller. The results are shown in Figure S3. There in the horizontal axis is depicted the level of naringenin production for each combination. In the vertical axis we plot the naringenin production with the perturbation relative to the unperturbed production. In this way, a point with 1 g L^{-1} in the horizontal axis and a 100% in the vertical would have the same level of production without and with the perturbation, note this would be an ideal situation.

As it is possible to see, the antithetic controller has more solutions in the upper part of the naringenin production (x-axis) than the direct controller. In a tuning stage it would be possible to select the parameters of the antithetic controller, corresponding to one of the solutions keeping large production values even under perturbations. Therefore, this controller will have a very good performance. In contrast, the direct controller has a big portion of its solutions grouped in the low producing end of the plot (low x-axis values), having an additional upper limit for the naringenin production with perturbation of 90%.

In Figure S4 we show the distribution of parameter values for the solutions with good performance

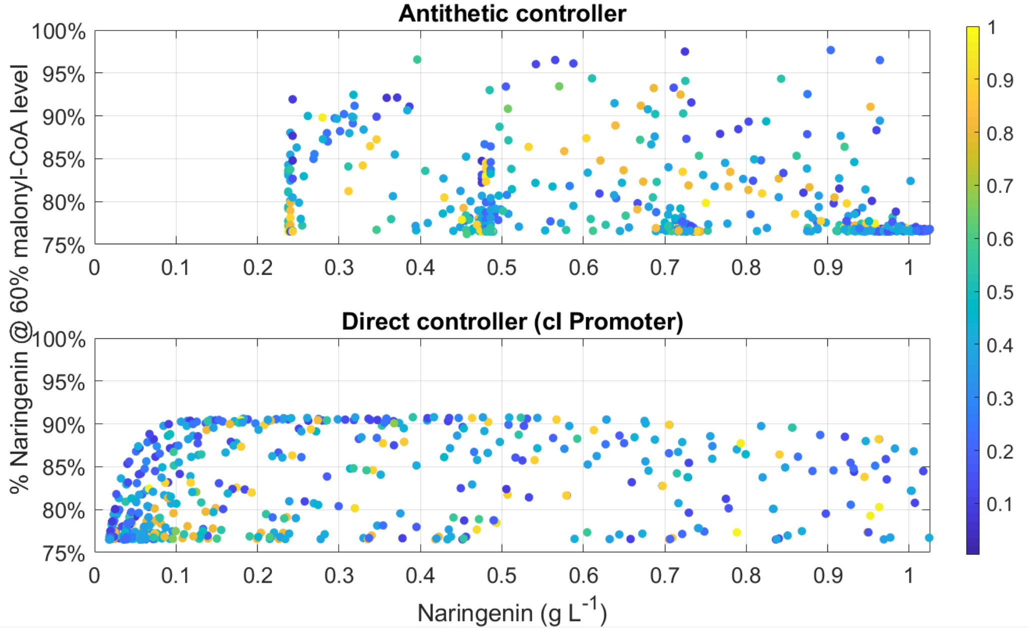

Figure S3: Performance comparison between the antithetic controller and the direct controller. The dots represent the production of certain parameter combination. The color of the dots, represent the distance to the nominal point (i.e. the one used in the main body of the paper for all the simulations giving a production of 1 g L^{-1}) where blue means short distance and yellow means big distance. Related to Figure 5.

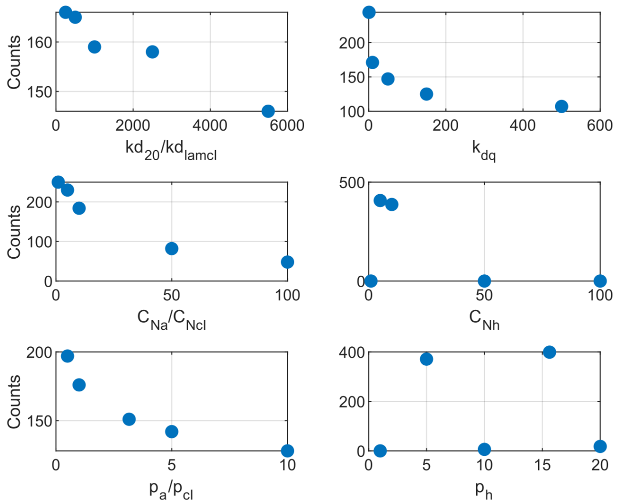

Figure S4: Number of solutions with good performance for each parameter value. Related to Figure 2.

(around 1 g L^{-1}) to see which parameter has a bigger influence. Here, we found that the copy number associated with most of the solutions with good performance are in the following ranges:

- The copy number of the plasmid of the biosensor (the one with the Pqdol promoter driving the expression of Anti$-\sigma$) 1 plasmid (250 solutions), 5 plasmids (230 solutions) and 10 plasmids (180

solutions). All together these three values represent more than 80% of the good solutions.

- The copy number of the plasmid of the actuator (the one with the P20 promoter driving the expression of CHS) 5 plasmid (407 solutions) and 10 plasmids (387 solutions). All together these two values represent 100% of the good solutions.

Supplemental References

Alberts, B., Bray, D., Hopkin, K., Johnson, A. D., Johnson, A., Roberts, K., Lewis, J., Raff, M., and Walter, P. (2009). *Essential Cell Biology*. (3rd ed.). Garland Science.

Anderson, J. C. (accessed April 22, 2020). Anderson promoter collection. MIT: Registry of Standard Biological Parts. `http://parts.igem.org/Promoters/Catalog/Anderson`.

Annunziata, F., Matyjaszkiewicz, A., Fiore, G., Grierson, C. S., Marucci, L., di Bernardo, M., and Savery, N. J. (2017). An orthogonal multi-input integration system to control gene expression in escherichia coli. *ACS Synthetic Biology*, *6*, 1816–1824.

Duigou, T., du Lac, M., Carbonell, P., and Faulon, J.-L. (2019). RetroRules: a database of reaction rules for engineering biology. *Nucleic Acids Research*, *47*, D1229–D1235.

Feher, T., Libis, V., Carbonell, P., and Faulon, J.-L. (2015). A Sense of Balance: Experimental Investigation and Modeling of a Malonyl-CoA Sensor in Escherichia coli. *Frontiers in Bioengineering and Biotechnology*, *3*, 46.

Michaelis, L., and Menten, M. (1913). Die kinetik der invertinwirkung biochem z 49: 333–369. *Find this article online*, .

Olsman, N., Baetica, A.-A., Xiao, F., Leong, Y. P., Murray, R. M., and Doyle, J. C. (2019). Hard Limits and Performance Tradeoffs in a Class of Antithetic Integral Feedback Networks. *Cell Systems*, *9*, 49–63.e16.

Rogers, J. K., Guzman, C. D., Taylor, N. D., Raman, S., Anderson, K., and Church, G. M. (2015). Synthetic biosensors for precise gene control and real-time monitoring of metabolites. *Nucleic Acids Research*, *43*, 7648–7660.

Rogers, J. K., Taylor, N. D., and Church, G. M. (2016). Biosensor-based engineering of biosynthetic pathways. *Current Opinion in Biotechnology*, *42*, 84–91.

Schomburg, I., Jeske, L., Ulbrich, M., Placzek, S., Chang, A., and Schomburg, D. (2017). The BRENDA enzyme information system–From a database to an expert system. *Journal of Biotechnology*, *261*, 194–206.

Takamura, Y., and Nomura, G. (1988). Changes in the intracellular concentration of acetyl-CoA and malonyl-CoA in relation to the carbon and energy metabolism of escherichia coli k12. *Microbiology*, *134*, 2249–2253.

Trabelsi, H., Koch, M., and Faulon, J.-L. (2018). Building a minimal and generalizable model of transcription factor–based biosensors: Showcasing flavonoids. *Biotechnology and Bioengineering*, *115*, 2292–2304.

Wu, J., Du, G., Chen, J., and Zhou, J. (2015). Enhancing flavonoid production by systematically tuning the central metabolic pathways based on a CRISPR interference system in escherichia coli. *Scientific Reports*, *5*.

Zhang, F., Carothers, J. M., and Keasling, J. D. (2012). Design of a dynamic sensor-regulator system for production of chemicals and fuels derived from fatty acids. *Nature Biotechnology*, *30*, 354–359.
